# Prognostic prediction of novel risk scores (AML-DRG and AML-HCT-CR) in acute myeloid leukemia patients with allogeneic hematopoietic stem cell transplantation

**DOI:** 10.1038/s41598-022-20735-1

**Published:** 2022-11-08

**Authors:** Weijie Cao, Xiaoning Li, Ran Zhang, Zhilei Bian, Suping Zhang, Li Li, Haizhou Xing, Changfeng Liu, Xinsheng Xie, Zhongxing Jiang, Xiaosheng Fang, Dingming Wan, Jifeng Yu

**Affiliations:** 1 Department of Hematology, the First Affiliated Hospital of Zhengzhou University, Zhengzhou, 450052 Henan China; 2 Department of Hematology, Shandong Provincial Hospital Affiliated to Shandong First Medical University, Jinan, 250098 Shandong China; 3 Henan International Joint Laboratory of Nuclear Protein Gene Regulation, Henan University College of Medicine, Kaifeng, 475004 Henan China

**Keywords:** Stem cells, Haematopoietic stem cells, Oncology, Cancer, Haematological cancer, Leukaemia, Myelodysplastic syndrome, Myeloma

## Abstract

We aimed to validate and prove the novel risk score models of acute myeloid leukemia (AML)-specific disease risk group (AML-DRG) and AML-Hematopoietic Cell Transplant-composite risk (AML-HCT-CR) in patients with acute myeloid leukemia (AML) after allogeneic hematopoietic stem cell transplantation (AHCT). Among the 172 AML patients analysed, 48.3% (n = 83) were females. Median age was 31.5 years (range 14 to 62 years), two patients was more than 60 years old (1.2%). Median follow-up was 44 months (range 1 to 94 months). According to the AML-DRG model, 109, 49 and 14 patients were in low-, intermediate- and high-risk group, respectively. According to the AML-HCT-CR model, 108, 30, 20 and 14 patients were in low-, intermediate-, high- and very high-risk group, respectively. Our results showed that the AML-DRG and AML-HCT-CR models significantly predicted cumulative incidence of relapse (*p* < 0.001; *p* < 0.001). But AML-DRG model was not associated with NRM (*p* = 0.072). Univariate analysis showed that the AML-DRG model could better stratify AML patients into different risk groups compared to the AML-HCT-CR model. Multivariate analysis confirmed that prognostic impact of AML-DRG and AML-HCT-CR models on post-transplant OS was independent to age, sex, conditioning type, transplant modality, and stem cell source (*p* < 0.001; *p* < 0.001). AML-DRG and AML-HCT-CR models can be used to effectively predict post-transplant survival in patients with AML receiving AHCT. Compared to AML-HCT-CR score, the AML-DRG score allows better stratification and improved survival prediction of AML patients post-transplant.

## Introduction

Acute myeloid leukemia (AML) is a clonal malignancy characterized by genetic heterogeneity due to recurrent gene mutations. Long term overall survival has stagnated in the past few decades. Even with an array of new gene mutation-targeted agents available for AML treatment^1^, the complete cure of leukemia still faces great challenges. Allogeneic hematopoietic stem cell transplantation (AHCT) is a curative treatment for AML patients^2,3^. Disease relapse and transplant-related mortality (TRM) are important for long-term survival of AML patients, especially the disease relapse^4,5^. How to prevent post-AHCT relapse on the basis of controlling the toxicities of conditioning regimen was important to improve the outcomes of AML patients^6,7^. Survival of patients after AHCT is largely dependent on disease- and patient-related factors^8–12^. Pre-transplant risk assessment is the key to optimizing transplant outcomes.

Several prognostic models have been developed in recent years. The disease risk index (DRI)^13,14^, representing disease-related factors, can predict overall survival (OS) but can’t integrate patient’s comorbidities and overall conditions. The hematopoietic cell transplantation-specific comorbidity index (HCT-CI) is also predictive of outcome but only includes comorbidities^15,16^. The disease risk comorbidity index (DRCI) and haplo-DRCI, integrating the DRI and HCT-CI, can effectively predict outcomes after AHCT^17^. Although previous studies have proved that minimal residual disease (MRD) is an independent predictor of survival in patients with AML^18–20^, MRD is excluded from most models. Therefore, two comprehensive new prognostic scores, AML-specific disease risk group (AML-DRG) and AML-Hematopoietic Cell Transplant-Composite Risk (AML-HCT-CR), have been shown to be predictive of OS and PFS in patients with AML received AHCT^21^. However, there are no published reports of the AML-DRG and AML-HCT-CR models in China. In this retrospective study, we aimed to verify the clinical effectiveness and generalizability of the AML-DRG and AML-HCT-CR models in a cohort of patients with AML receiving AHCT.

## Materials and methods

### Data source

A total of 172 adult patients diagnosed with non-M3 acute myeloid leukemia who underwent the first AHCT from January 1, 2013 to December 30, 2018 in the First Affiliated Hospital of Zhengzhou University and Shandong Provincial Hospital Affiliated to Shandong First Medical University were enrolled in this study. Secondary AML was defined as AML developed after treatment with systemic chemotherapy and/or radiation therapy. All enrolled subjects in this study provided written informed consent. The study was conducted in accordance with the Declaration of Helsinki. All patients were followed up through our outpatient clinic, medical records in hospital, or by telephone cells. The follow-up endpoint was December 30, 2020.

### Rating scales

AML-DRG score assignment: 1 for secondary AML, 1 for adverse European Leukemia 2017 genetic risk (ELN2017 genetic risk), 2 for CR with MRD positive or unknown MRD status, 4 for active disease^21^. The AML-HCT-CR score assignment: AML-DRG score with the addition of 1 score for age ≥ 60 and 1 score for HCT-CI ≥ 3^21^. DRI was applied as described by Armand et al.^13^. HCT-CI and HCT-CI/Age were applied as published by Sorror et al.^15,16^. ELN2017 genetic risk group was applied as described by Döhner, et al.^22^. MRD was assessed pre-HSCT by flow cytometry. MRD < 0.1% was judged to be negative^23^.

### Transplant protocol

All patients received myeloablative conditioning. The major conditioning regimen for the identical sibling donor (ISD) group as follows: hydroxyurea (40 mg/kg/12 h, day − 10), cytarabine (1 ~ 1.5 g/m^2^/day, day − 9), busulfan (0.8 g/kg/6 h, days − 8 ~ − 6), cyclophosphamide (1.8 g/m^2^/day, days − 5 and − 4). For HLA-haploidentical and HLA-matched unrelated donor transplants, the major conditioning regimen as follows: cytarabine (4 g/m^2^/day, days − 10 and − 9), busulfan (0.8 g/kg/6 h, days − 8 to − 6), cyclophosphamide (1.8 g/ m^2^/day, days − 5 and − 4), anti-thymocyte globulin (ATG) (2.5 mg/kg/day, days − 5 to − 2). Four patients received total body irradiation (TBI)-based regimen: cyclophosphamide (60 mg/kg/day, day − 6 and − 5) , total body irradiation (12 to 14 Gy, day − 3 ~ − 1). The GVHD prevention scheme used cyclosporine combined with mycophenolate mofetil and methotrexate.

### Outcomes

The primary endpoint was overall survival (OS) at 3 years after transplantation. The secondary endpoints were progression-free survival (PFS) at 3 years, non-relapse mortality (NRM) at 3 years, and cumulative incidence of relapse at 3 years. OS was defined as time from transplantation until death from any cause. PFS was defined as time from randomization to disease progression. Non-relapse mortality was defined as death from any cause not subsequent to relapse. Relapse was defined as either reappearance of leukemic blasts in the peripheral blood or at least 5% blasts in the bone marrow aspirate or biopsy specimen not attributable to any other cause, or reappearance or new appearance of extramedullary leukemia. Acute GVHD and chronic GVHD were diagnosed and graded according to the standard international criteria^24,25^.

### Statistical analysis

OS, PFS were estimated using the Kaplan–Meier method and compared using the Log-rank test. Cumulative incidences of relapse, non-relapse mortality, and GVHD were calculated by accounting for competing risks. Competing risks for GVHD included death without GVHD and relapse. Relapse was a competing risk for non-relapse mortality, and non-relapse mortality was a competing risk for relapse. The comparison of the cumulative incidence in the presence of a competing risk was done using the Fine and Gray model. The impact of the AML-DRG and AML-HCT-CR models on survival outcomes were determined using univariable and multivariable Cox proportional hazards regression models. The discriminative ability of the models was assessed by Harrell’s C-statistics. p < 0.05 was considered significant. SPSS version 23.0 and R version 3.6.2 were used for data analysis.

### Ethics approval and consent to participate

Informed consent was obtained for study participation from all patients, parent and/or legal guardian for minors and the protocol was approved by the Ethics Committee of the First Affiliated Hospital of Zhengzhou University.

## Results

### Patient characteristics

Among the 172 AML patients analysed, 48.3% (n = 83) were females. Median age was 31.5 years (range 14 to 62 years), two patients was more than 60 years old (1.2%). Median follow-up was 44 months (range 1 to 94 months). According to the AML-DRG model, 109, 49 and 14 patients were in low-, intermediate- and high-risk group, respectively. According to the AML-HCT-CR model, 108, 30, 20 and 14 patients were in low-, intermediate-, high- and very high-risk group, respectively. The basic clinical data of the patients were shown in Table 1.

**Table 1:** Patient characteristics.

Characteristic	All patients	
	No	%
No. of patients	172	
Female sex	83	48.3
Median age, years	31.5 (14–62)	
White blood cells at diagnosis, × 109 per L	21.6 (0.6–469.3)	
**AML type (%)**		
De novo	165	95.9
Secondary	7	4.1
**ELN2017 genetic risk group**		
Favorable	14	8.1
Intermediate	88	51.2
Adverse	70	40.7
**MRD status at transplant**		
CR with MRD negative	46	26.7
CR with MRD positive	101	58.7
Active disease	25	14.5
Median HCT-CI/Age	1 (0–5)	
**DRI (%)**		
Low	6	3.5
Intermediate	120	69.8
High	43	25.0
Very high	3	1.7
**Conditioning type (%)**		
Busulfan-based	166	96.5
TBI-based	6	3.5
**Donor type (%)**		
HLA-matched sibling donor	81	47.1
HLA-matched unrelated donor	19	11.0
HLA-haploidentical donor	72	41.9
**Stem cell source (%)**		
PBSC	161	93.6
PBSC + BMSC	11	6.4

*AML* acute myeloid leukemia, *CR* complete remission, *HC* CI/Age-comorbidity-age index, *DRI* disease risk index, *BMSC* bone marrow stem cell, *PBSC* peripheral blood stem cell, *TBI* total body irradiation.

### GVHD

Among the 172 patients, grade II to IV acute GVHD developed in 39 patients (22.7%) and chronic GVHD developed in 38 patients (22.1%). For the entire cohort, the cumulative incidence of grade II to IV acute GVHD at day 100 was 22.7% (95% CI 20.0–25.4), the cumulative incidence of all-grade chronic GVHD at 2 years was 21.7% (95% CI 19.0–24.4). The cumulative 100-day incidence of grade II to IV acute GVHD for the low-, intermediate- and high-risk AML-DRG groups was 22.0% (95% CI 18.3–25.7), 18.4% (95% CI 11.8–25.0) and 7.1% (95% CI 0–21.1), differences are not statistically significant ( *p* = 0.605). The incidence for all-grade chronic GVHD at 2 years was 23.2% (95% CI 19.4–27.0), 22.4% (95% CI 15.5–29.3) and 7.1% (95% CI 0–22.1), respectively (*p* = 0.430) (Table 2).

**Table 2:** Outcomes of AHCT according to the AML-DRG and AML-HCT-CR models.

	3-year OS,	3-year PFS,	3-year relapse,	3-year NRM,	Grade II–IV aGVHD, %	ALL-grade cGVHD, %
	% (95%CI)	% (95%CI)	% (95%CI)	% (95%CI)	(95%CI)	(95%CI)
**AML-DRG**	*P* < 0.001	*P* < 0.001	*P* < 0.001	*P* = 0.072	*P* = 0.605	*P* = 0.430
Low (n = 109)	69.6 (61.0–79.6)	67.3 (58.8–77.2)	12.2 (9.5–16.3)	18.8 (15.1–22.5)	22.0 (18.3–25.7)	23.2 (19.4–27.0)
Intermediate (n = 49)	38.6 (27.1–55.0)	34.5 (23.4–50.8)	32.7 (25.4–40.0)	32.9 (25.6–40.2)	18.4 (11.8–25.0)	22.4 (15.5–29.3)
High (n = 14)	7.1 (1.1–47.2)	7.1 (1.1–47.2)	50.0 (30.2–69.8)	42.9 (22.9–62.9)	7.1 (0–21.1)	7.1 (0–22.1)
**AML-HCT-CR**	*P* < 0.001	*P* < 0.001	*P* < 0.001	*P* = 0.008	*P* = 0.582	*P* = 0.287
Low (n = 108)	69.4 (60.7–79.4)	67.1 (58.5–77.0)	12.3 (8.9–15.7)	19.0 (15.3–22.7)	21.3 (17.5–25.1)	23.4 (19.6–27.2)
Intermediate (n = 30)	59.6 (44.3–80.2)	52.9 (37.6–74.4)	26.7 (16.4–37.0)	20.4 (10.5–30.3)	13.3 (4.3–22.3)	30.3 (19.8–40.8)
High (n = 20)	10.0 (2.7–37.2)	10.0 (2.7–37.2)	45.0 (30.1–59.9)	45.0 (29.9–60.1)	30.0 (15.8–44.2)	10.0 (0–21.7)
Very high (n = 14)	7.1 (1.1–47.2)	7.1 (1.1–47.2)	42.9 (23.4–62.4)	50.0 (30.0–70.0)	7.1 (0–21.1)	7.1 (0–22.7)

*OS* overall survival, *PFS* progression-free survival, *NRM* non-relapse mortality, *aGVHD* acute GVHD, *cGVHD* chronic GVHD.

For the AML-HCT-CR model, the cumulative 100-day incidence of grade II to IV acute GVHD for the low-, intermediate-, high- and very high-risk groups was 21.3% (95% CI 17.5–25.1), 13.3% (95% CI 4.3–22.3), 30.0% (95% CI 15.8–44.2) and 7.1% (95% CI 0–21.1), respectively ( *p* = 0.582). The incidence for all-grade chronic GVHD at 2 years was 23.4% (95% CI 19.6–27.2), 30.3% (95% CI 19.8–40.8), 10.0% (95% CI 0–21.7) and 7.1% (95% CI 0–22.7), respectively ( *p* = 0.287) (Table 2).

### Relapse and NRM

For the entire cohort, the 3-year cumulative incidences of relapse and NRM were 21.1% (95% CI 18.5–23.7) and 24.8% (95% CI 22.1–27.5), respectively. We found that relapse and NRM occurred in 37 (21.5%) and 44 (25.6%) of 172 patients. Reasons for relapse were hematological in 30 patients (17.4%), extramedullary in 4 patients (2.3%), and hematological plus extramedullary in 5 patients (2.9%).

**Figure 1:**
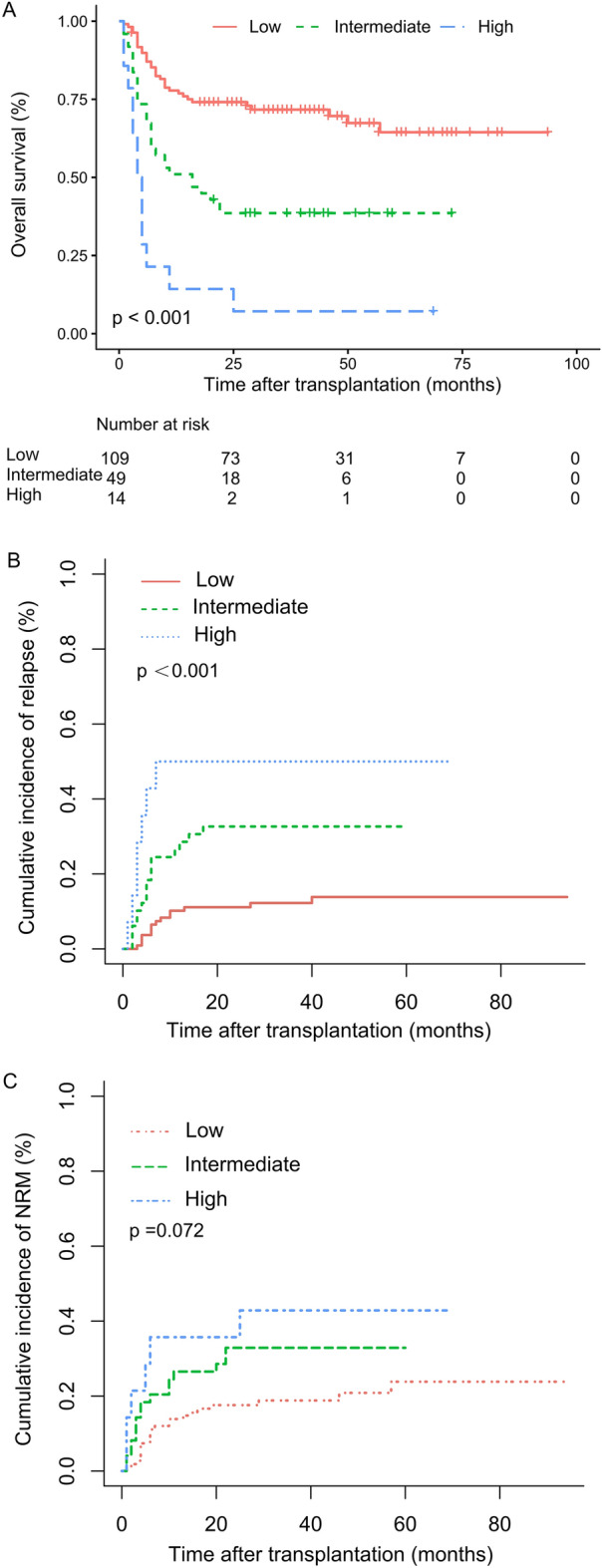
Comparison of overall survival (**A**), cumulative incidence of relapse (**B**) and cumulative incidence of NRM (**C**) by the AML-DRG model in patients with low-, intermediate- and high-risk groups.

**Figure 2:**
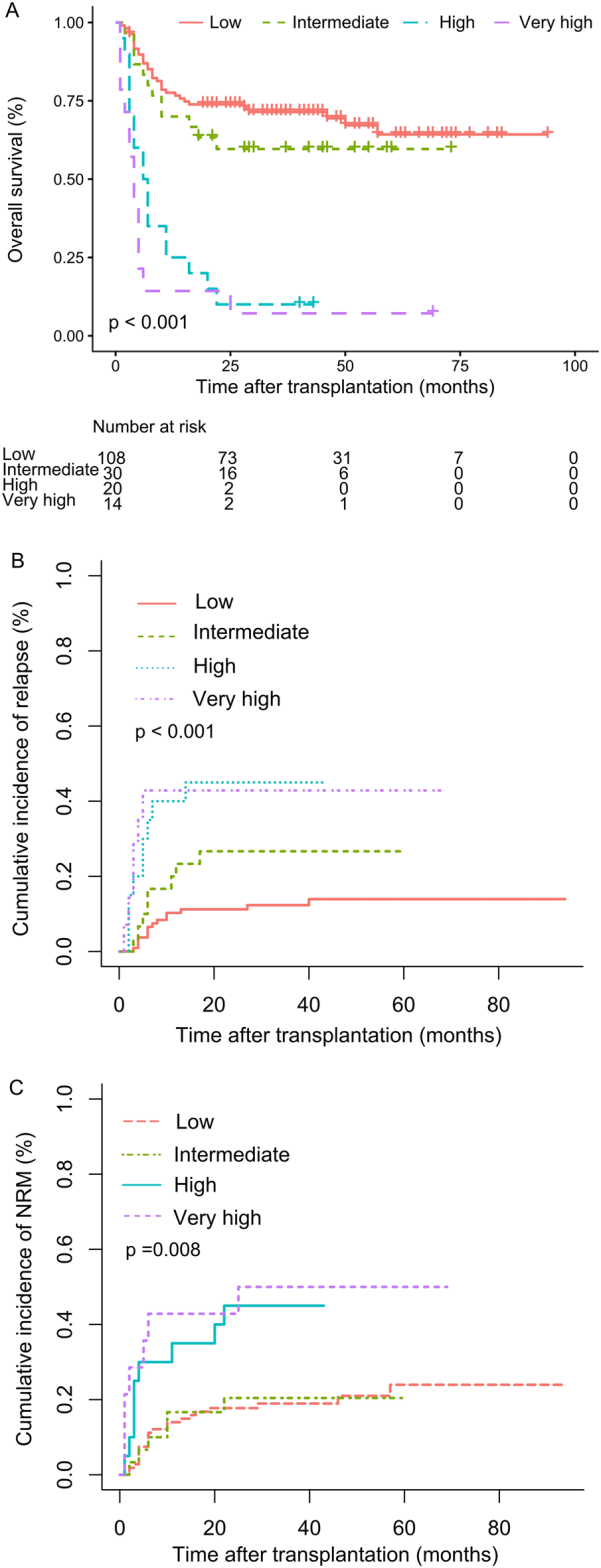
Comparison of overall survival (**A**), cumulative incidence of relapse (**B**) and cumulative incidence of NRM (**C**) by the AML-HCT-CR model in patients with low-, intermediate- and high-risk groups.

The 3-year cumulative incidence of relapse for the low-, intermediate- and high-risk AML-DRG groups was 12.2% (95% CI 9.5–16.3), 32.7% (95%CI 25.4–40.0) and 50.0% (95% CI 30.2–69.8), respectively (*p* < 0.001) (Fig. 1B) , with the corresponding 3-year NRM was 18.8% (95% CI 15.1–22.5), 32.9% (95% CI 25.6–40.2) and 42.9% (95% CI 22.9–62.9), differences are not statistically ( *p* = 0.072) (Fig. 1C). The 3-year cumulative incidence of relapse for the low-, intermediate-, high- and very high-risk AML-HCT-CR groups was 12.3% (95% CI 8.9–15.7), 26.7% (95% CI 16.4–37.0), 45.0% (95% CI 30.1–59.9) and 42.9% (95% CI 23.4–62.4), respectively (*p* < 0.001) (Fig. 2B), with the corresponding 3-year NRM was 19.0% (95% CI 15.3–22.7), 20.4% (95% CI 10.5–30.3), 45.0% (95% CI 29.9–60.1) and 50.0% (95% CI 30.0–70.0), respectively (*p* = 0.008) (Fig. 2C).

### OS and PFS

The 3-year OS and PFS of the entire cohort were 56.9% (95% CI 49.9–64.9) and 53.1% (95% CI 45.9–61.3). Patients in low-, intermediate- and high-risk AML-DRG groups had median OS of 33.0 (1 ~ 94), 16.0 (1 ~ 73) and 4.5 (1 ~ 69) months, respectively (p < 0.001), with the corresponding 3-year OS of 69.6% (95% CI 61.0–79.6), 38.6% (95% CI 27.1–55.0) and 7.1% (95% CI 1.1–47.2), respectively (p < 0.001). The 3-year PFS were 67.3% (95% CI 58.8–77.2), 34.5% (95% CI 23.4–50.8) and 7.1% (95% CI 1.1–47.2), respectively (p < 0.001) (Fig. 1A). The median OS for the low-, intermediate-, high- and very high-risk AML-HCT-CR groups were 33.0 (1 ~ 94), 28.9 (2 ~ 73), 6.5 (1 ~ 43) and 4.0 (1 ~ 69) months, respectively (p < 0.001), with the corresponding 3-year OS of 69.4% (95% CI 60.7–79.4), 59.6% (95% CI 44.3–80.2), 10.0% (95% CI 2.7–37.2) and 7.1% (95% CI 1.1–47.2), respectively (p < 0.001) (Fig. 2A). The 3-year PFS were 67.1% (95% CI 58.5–77.0), 52.9% (95% CI 37.6–74.4), 10.0% (95% CI 2.7–37.2) and 7.1% (95% CI 1.1–47.2), respectively (p < 0.001) (Table 2).

In univariable analysis for OS, patients with intermediate and high-risk AML-DRG groups had a significantly increased risk of death with hazard ratio (HR) of 2.62 (95% CI 1.60–4.31; *p* < 0.001) and 7.08 (95% CI 3.68–13.63; *p* < 0.001), respectively when compared with the low-risk group. Also, the risk of death was higher in high-risk group compared with the intermediate-risk group (HR 2.63, 95% CI 1.35–5.10; *p* = 0.004), confirming the ability of the AML-DRG model in post-transplant survival prediction. For AML-HCT-CR groups, patients with high (HR 5.44, 95% CI 3.03–9.78; *p* < 0.001) and very high-risk groups (HR 8.35, 95% CI 4.32–16.14; *p* < 0.001) had significantly increased risk of death than low-risk group, while there was no difference between low and intermediate-risk (HR 1.39, 95% CI 0.72–2.69; *p* = 0.330) and between high and very high group (HR 1.53,95% CI 0.74–3.17; *p* = 0.251). Similar results were found in a univariable analysis for PFS as summarized in Table 3.

**Table 3:** Univariable analysis evaluating the impact of the HCT-CR model on OS and PFS.

Univariable analysis for OS	HR	95%CI	*P* value
**AML-DRG**			
Low	Reference		
Intermediate	2.62	1.60–4.31	< 0.001
High	7.08	3.68–13.63	< 0.001
**AML-HCT-CR**			
Low	Reference		
Intermediate	1.39	0.72–2.69	0.330
High	5.44	3.03–9.78	< 0.001
Very high	8.35	4.32–16.14	< 0.001
**Univariable analysis for PFS**			
AML-DRG			
Low	Reference		
Intermediate	2.70	1.67–4.35	< 0.001
High	6.78	3.55–12.93	< 0.001
**AML-HCT-CR**			
Low	Reference		
Intermediate	1.54	0.83–2.86	0.171
High	5.44	3.06–9.67	< 0.001
Very high	7.72	4.04–14.74	< 0.001

*HR* hazard ratio, *CI* confidence interval, *OS* overall survival, *PFS* progression-free survival.

### Multivariate analysis

Multivariable analysis confirmed that the HCT-CR and AML-HCT-CR models could be used to predict the OS of patients in different risk groups after adjusted for other variables including age, sex, conditioning type, transplant modality, and stem cell source. See Table 4 for details.

**Table 4:** Multivariable analysis for OS.

Variable	AML-DRG			AML-HCT-CR		
	HR	95%CI	*P* value	HR	95%CI	*P* value
**AML-DRG or AML-HCT-CR**			< 0.001			< 0.001
Low	Reference			Reference		
Intermediate	2.77	1.67–4.57	< 0.001	1.53	0.78–3.00	0.212
High	7.23	3.65–14.30	< 0.001	4.77	2.60–8.75	< 0.001
Very high				8.41	4.26–16.60	< 0.001
Age (continuous variable)	1.02	1.00–1.04	0.101	1.01	0.99–1.03	0.285
Sex (male vs. female)	0.81	0.59–1.52	0.810	0.92	0.57–1.49	0.736
Conditioning type (Bu- vs. TBI-based)	1.60	0.62–4.15	0.336	1.27	0.48–3.33	0.633
PBSC vs. BMSC + PBSC	1.64	0.73–3.70	0.232	1.47	0.64–3.39	0.364
**Transplant modality**						
HLA-matched sibling donor	Reference			Reference		
HLA-matched unrelated donor	0.49	0.17–1.42	0.189	0.60	0.21–1.76	0.355
HLA-haploidentical donor	1.16	0.70–1.91	0.568	1.12	0.68–1.86	0.652

*HR* hazard ratio, *CI* confidence interval, *BMSC* bone marrow stem cell, *PBSC* peripheral blood stem cell, *Bu* Busulfan, *TBI* total body irradiation.

### Comparison of prognostic stratification

The C-indexes of the AML-DRG, AML-HCT-CR, DRI, ELN2017 genetic risk, and HCT-CI/Age model were 0.69 (95% CI 0.61–0.78), 0.71 (95% CI 0.63–0.79), 0.61 (95% CI 0.52–0.69), 0.52 (95% CI 0.43–0.60) and 0.59 (95% CI 0.50–0.67), respectively. Compared with the DRI model and the HCT-CI/Age, the AML-DRG and AML-HCT-CR models had significantly better discrimination ability on OS prediction with C-index. The risk assessment ability of AML-DRG and AML-HCT-CR may be better than that of ELN2017 genetic risk, DRI and HCT-CI/Age models.

## Discussion

Risk stratification is essential to predict the prognosis of patients with AML receiving AHCT. Recently, the AML-DRG and AML-HCT-CR models, which combines DRI, HCT-CI, ELN2017 risk classification, MRD and other important prognostic factors, was published and has demonstrated a significant impact in terms of OS and PFS^21^. In this study, we examined the effect of the AML-DRG and AML-HCT-CR models on clinical outcomes of AHCT. The results demonstrated that both AML-DRG and AML-HCT-CR models could significantly predict the OS, PFS and relapse. The AML-HCT-CR model could significantly predict the NRM. While the GVHD did not reach statistical significance.

In our retrospective study including 172 patients with AML, we confirmed that the AML-DRG and AML-HCT-CR models have a prognostic prediction on OS and PFS (all p < 0.001). This is consistent with the reference publication. Kongtim et al.^21^ reported that the OS at 5 years for low-, intermediate- and high-risk AML-DRG risk groups were 62.8%, 33.1% and 12.6%, respectively, and 5-year PFS were 60.4%, 31.1%, and 7.9%, respectively. The OS at 5 years for low-, intermediate-, high- and very high-risk AML-HCT-CR risk groups were 71.1%, 53.6%, 37.4%, and 12.7%, respectively, the corresponding PFS at 5 years were 67.4%, 51.7%, 36.2%, and 9.6%, respectively. We also performed pairwise comparisons in survival analysis among different groups. The results showed that for AML-DRG, all pairwise comparisons were statistically significant. However, unlike the reference publication, we did not find the difference of OS between AML-HCT-CR low-risk and intermediate-risk group (69.4% vs 59.6%, p = 0.330), high-risk and very high-risk group (10.0% vs 7.1%, p = 0.251). Fist, the sample of high- and very high-risk AML-HCT-CR patients was relatively small. Second, in our study, the conditioning regimens included busulfan (Bu)- and total body irradiation (TBI)-based regimens in patients, while the reference publication included busulfan (Bu)- and Melphalan-based regimens. Third, unlike the reference publication, only two patients in our sample were older than 60 years. Perhaps these reasons caused our results to be different from the reference publication. Therefore, a multi-centre clinical trial is required to confirm the findings from the reference publication. The multivariable analyses have shown that prognostic prediction of the AML-DRG and AML-HCT-CR models on post-transplant survival was independent to age, sex, conditioning type, transplant modality, and stem cell source (p < 0.001; p < 0.001). This means that the model can be applied in patients transplanted using both HLA-matched and unmatched donors.

Since the AML-DRG and AML-HCT-CR models have been developed and validated their utility in mortality prediction, we further identified the specific cause of mortality. Our study found that the AML-DRG and AML-HCT-CR models were associated with the 3-year OS, mainly due to relapse. The AML-DRG model categorized patients into 3 distinct relapse risk groups, with 3-year cumulative incidence of relapse ranging between 12.2% for the low-risk group to 50.0% for the high-risk group. Similar results were found for the AML-HCT-CR model (12.3%for the low-risk group and 42.9% for the very high-risk group). The results suggest that for the patients in the intermediate and high-risk groups of AML-DRG and AML-HCT-CR models, MRD and other related indexes should be closely monitored after transplantation, and maintenance treatment or pre-emptive treatment should be given, so as to reduce the risk of recurrence and improve the efficacy of the transplantation. Patients in the very high-risk AML-HCT-CR group had higher recurrence and NRM rates, especially high NRM rates (42.9%for relapse rate and 50.0% for NRM rate). These patients usually had advanced-stage disease and/or high comorbidities burden before AHCT, which suggested that they had a higher risk of disease relapse and may be vulnerable to drug toxicities and transplant complications. A lower intensity conditioning regimen may help to prevent the transplant-related toxicity and mortality. However, this may lead to high relapse rates after AHCT, particularly for those with relapse/refractory leukemia^6,7^. Therefore, how to prevent relapse after AHCT on the basis of controlling the toxicity of the conditioning regimen is important to improve the clinical outcome of patients with very high-risk AML-HCT-CR group.

Our study found that the AML-DRG and AML-HCT-CR models had a significantly better discrimination ability on OS prediction with Harrell C-index of0.69 and 0.71, respectively, when compared with the DRI model (C-index0.61), HCT-CI/Age (C-index0.59) and ELN2017 risk classification (C-index0.52). This was partially in accordance with the reference study in which the AML-DRG and AML-HCT-CR models significantly better predicted risk of death after transplant with C-indices of 0.672 and 0.715, respectively^21^. It demonstrated that AML-DRG and AML-HCT-CR models provide better tools for risk stratification of patients.

The AML-DRG model had no prognostic for NRM. In addition, both AML-DRG and AML-HCT-CR models were not prognostic for GVHD. Those data are also very important for HSCT choice. Further incorporation of NRM- and GVHD- related specificity indicators may help to achieve a more comprehensive scoring system. Although HCT-CI had been considered be prognostic of NRM and GVHD^16,26,27^, we did not find AML-HCT-CR be prognostic for GVHD. A possible explanation for our results is that combining AML-DRG and HCT-CI probably weakens the weight of HCT-CI in GVHD prognostication.

## Conclusions

In conclusion, our data confirm results similar to the reference publication and provide useful information on OS, PFS, relapse, and NRM prediction. Compared to AML-HCT-CR model, the AML-DRG allows better stratification and improved survival prediction of AML patients post-transplant. Considering the absence of prognosis for NRM and GVHD, we recommend using the AML-DRG and HCT-CI separately to obtain more accurate and relevant information to guide transplant choice.

## Data Availability

Data and material will be available upon corresponding author approval. All data sets generated/analysed for this study are included in the manuscript and the additional files.

## Abbreviations

aGVHD: Acute GVHD
AHCT: Allogeneic hematopoietic stem cell transplantation
AML: Acute myeloid leukemia
AML-DRG: AML-specific disease risk group
AML-HCT-CR: AML-Hematopoietic Cell Transplant-composite risk
ATG: Anti-thymocyte globulin
BMSC: Bone marrow stem cell
Bu: Busulfan
cGVHD: Chronic GVHD
CI: Confidence interval
CR: Complete remission
DRCI: Disease risk comorbidity index
DRI: Disease risk index
ELN2017 genetic risk: European Leukemia Net 2017 genetic risk
HCT-CI: Hematopoietic cell transplantation-specific comorbidity index
HR: Hazard ratio
ISD: Identical sibling donor
MRD: Minimal residual disease
NRM: Non-relapse mortality
OS: Overall survival
PBSC: Peripheral blood stem cell
PFS: Progression-free survival
TBI: Total body irradiation
TRM: Transplant-related mortality
